# FAM84B, amplified in pancreatic ductal adenocarcinoma, promotes tumorigenesis through the Wnt/β-catenin pathway

**DOI:** 10.18632/aging.103044

**Published:** 2020-04-14

**Authors:** Xin Zhang, Jiapeng Xu, Ronglin Yan, Yu Zhang, Zunqi Hu, Hongbing Fu, Qing You, Qingping Cai, Dejun Yang

**Affiliations:** 1 Department of Gastrointestinal surgery, Changhai Hospital, Second Military Medical University, Yangpu 200433, Shanghai, China; 2 Department of Gastrointestinal Surgery, Changzheng Hospital, Second Military Medical University, Huangpu 200003, Shanghai, China

**Keywords:** proliferation, apoptosis, glycolysis, Wnt/β-catenin, gemcitabine

## Abstract

Altered expression of family with sequence similarity 84, member B (FAM84B) has been found in various human cancers. However, the expression and function of FAM84B in pancreatic ductal adenocarcinoma (PDAC) has not been studied. Here, by analyzing The Cancer Genome Atlas cohort, we found that *FAM84B* amplification was observed in 11% of 141 PDAC patients, and *FAM84B* amplification was correlated with higher mRNA expression of FAM84B. *FAM84B* amplification and overexpression was significantly correlated with poor overall survival. Moreover, knockdown of FAM84B in PDAC cell lines suppressed cell proliferation and induced apoptosis. FAM84B knockdown also suppressed mitochondrial function and glycolysis of PDAC cells. Interestingly, knockdown of FAM84B decreased the nuclear accumulation of β-catenin, and the expression of c-Myc and lactate dehydrogenase A, but enhanced the expression of Survivin. On the contrary, FAM84B overexpression displayed reversed effects in cell proliferation, apoptosis, mitochondrial function, and glycolysis, which was blocked by the Wnt/β-catenin pathway inhibitor (XAV939). In addition, PDAC cells with lower expression of FAM84B were more sensitive to gemcitabine-induced cell proliferation inhibition both *in vitro* and *in vivo*. In conclusion, FAM84B plays an important role in aerobic glycolysis and tumorigenesis in PDAC and Wnt/β-catenin may be involved in this process.

## INTRODUCTION

Pancreatic ductal adenocarcinoma (PDAC) is a highly lethal human malignancy with an extremely poor five-year survival rate [1]. The risk factors of PDAC include smoking, diabetes, obesity, and pancreatitis [2]. In recent years, important advances have been achieved in the understanding of the epigenetic and genetic alterations in PDAC development and metastasis [3–5], and molecular pathways that drive the formation and development of PDAC have been elucidated, such as Notch [6], mitogen-activated protein kinase (MAPK) [7], transforming growth factor (TGF)-β [7], Hedgehog [8], and Wnt/β-catenin pathways [8]. However, the prognosis of PDAC remains poor because minimal progress has been made in early diagnosis, prevention, and treatment of patients with PDAC [9–11]. More than 80% of PDAC patients have advanced disease when first diagnosed [1]. Gemcitabine remains the standard agent for the treatment of advanced PDAC, but acquired resistance within weeks of chemotherapy initiation limits the treatment potency and leads to the poor prognosis [12]. Research on the molecular basis of gemcitabine resistance may help increase the efficacy of chemotherapy and improve the clinical outcomes.

Family with sequence similarity 84, member B (FAM84B), also known as NSE2, is located on chromosome 8q24.21 [13], where pancreatic cancer susceptibility region has been identified [14]. Accumulated evidence has supported the association between FAM84B and carcinogenesis. FAM84B was overexpressed in breast cancer [13], prostate cancer [15], esophageal squamous cell carcinoma (ESCC) [16, 17], epithelial ovarian cancer [18], and colorectal cancer [19], but significantly reduced in gastroesophageal junction adenocarcinomas [20]. Moreover, FAM84B knockdown in ESCC cells significantly reduced *in vitro* cell growth, migration, and invasion [17], and delayed *in vivo* tumor growth [16]. FAM84B overexpression in prostate cancer cells significantly enhanced *in vitro* cell invasion and the growth of xenografts and lung metastasis [15, 21]. However, little attention has been focused on the possible functions of FAM84B in PDAC.

Here, we discovered that the amplification and elevated expression of FAM84B in human PDAC specimens were closely related to the overall survival of patients. FAM84B expression was correlated with proliferation, apoptosis, aerobic glycolysis, and gemcitabine resistance of PDAC cell lines. We further found that the Wnt/β-catenin pathway might be involved in the functions of FAM84B during pancreatic carcinogenesis. Our current study may provide new insights into the potential mechanisms of PDAC pathogenesis and the development of novel therapy targets for PDAC.

## RESULTS

### FAM84B amplification in patients with PDAC

**Figure 1:**
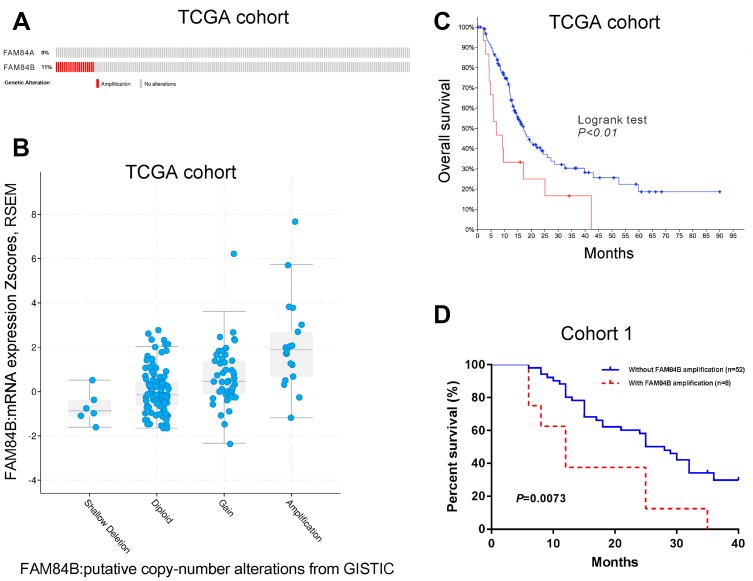
**FAM84B amplification in PDAC.** (**A**) CNV analysis of FAM84A and FAM84B in TCGA PDAC dataset (n=141). (**B**) *FAM84B* amplification was associated with higher mRNA expression of FAM84B in TCGA PDAC dataset. (**C**) Kaplan-Meier survival analysis of TCGA PDAC dataset suggested that *FAM84B* amplification indicated worse prognosis. (**D**) Kaplan-Meier survival analysis of cohort 1 patients.

Data from The Cancer Genome Atlas (TCGA, https://tcga-data.nci.nih.gov/tcga) on pancreatic ductal adenocarcinoma (PDAC) indicated *FAM84B* amplification in 11% of 141 PDAC patients, while no amplification was observed for *FAM84A* (Figure 1A). Moreover, TCGA data also suggested that *FAM84B* amplification was correlated with higher mRNA expression of FAM84B (Figure 1B), and predicted poorer prognosis in PDAC (Figure 1C).

*FAM84B* amplification using real-time PCR analysis was seen in 8/60 (13.3%) (*FAM84B* gene copy numbers (GCN): 4-6) of cohort 1 patients form our hospital. Kaplan-Meier survival curves and log-rank analysis showed that PDAC patients with *FAM84B* amplification in cohort 1 had shorter survival time (P <0.01, Figure 1D).

### FAM84B expression in patients with PDAC

**Figure 2:**
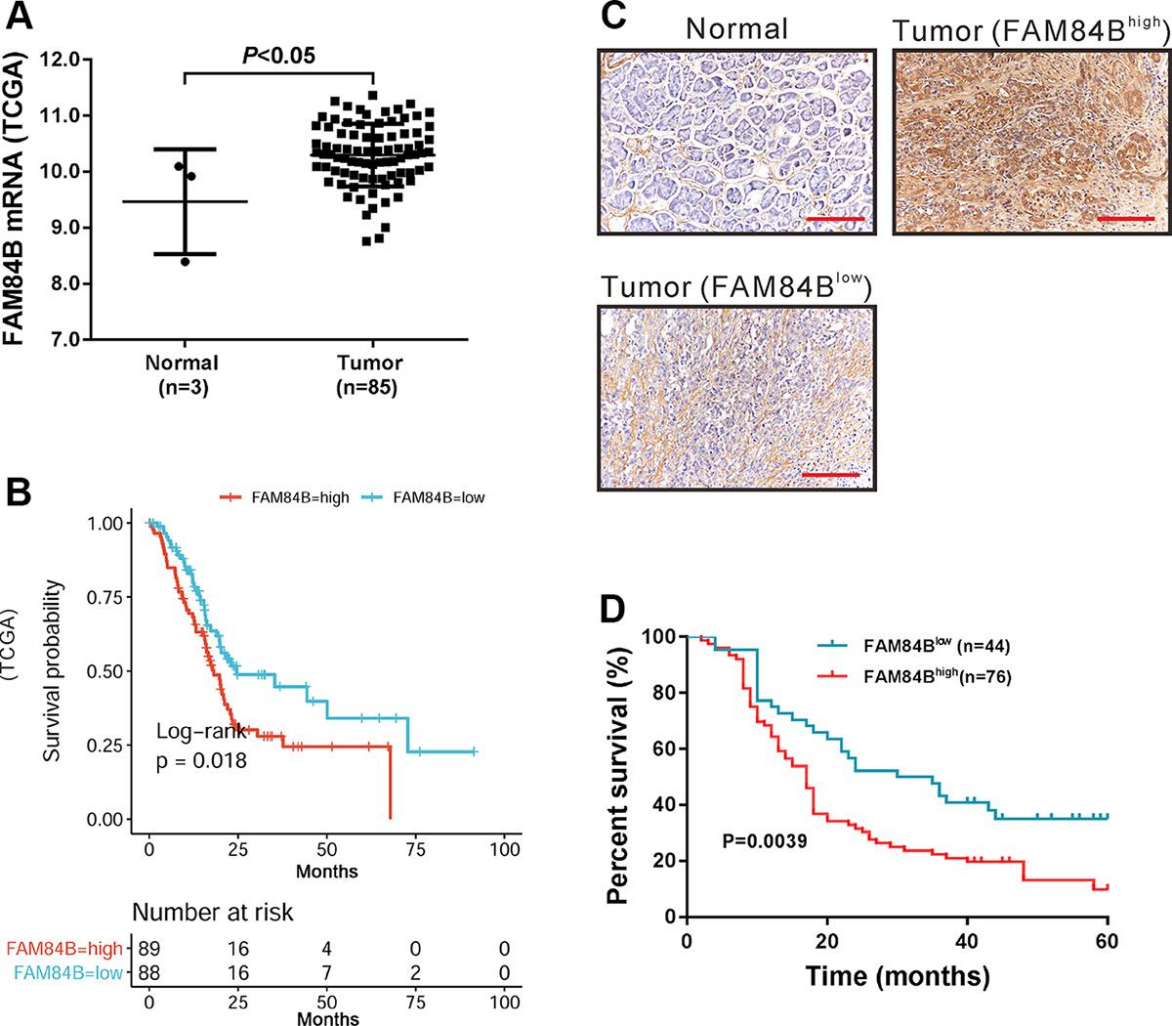
**FAM84B expression in PDAC.** (**A**) mRNA expression analysis of FAM84B in TCGA PDAC dataset. (**B**) Survival analysis of FAM84B in TCGA PDAC dataset. High FAM84B expression indicated worse prognosis. (**C**) IHC analysis of FAM84B expression in PDAC tissues and adjacent normal tissues (magnification scale bar, 100 μm) from cohort 2 patients. (**D**) Survival analysis of PDAC based on IHC analysis.

Data from TCGA indicated that FAM84B mRNA expression was up-regulated in PDAC tissues (Figure 2A). Moreover, TCGA data also suggested that FAM84B overexpression was correlated with poorer prognosis in PDAC (Figure 2B).

FAM84B protein expression was then analyzed in cohort 2 patients (n=120) by IHC staining. The results showed that FAM84B protein expression was high in 76 cases (63.3%, Figure 2C). Chi-square test or Fisher exact test indicated that FAM84B expression was strongly correlated with tumor size, tumor differentiation, and lymph node status (Table 1). Kaplan-Meier survival curves and log-rank analysis showed that higher expression of FAM84B was associated with shorter survival time in patients with PDAC (P <0.01, Figure 2D).

**Table 1:** Clinicopathological features and correlation of FAM84B expression in patients with PDAC (n=120).

		**FAM84B^high^**	**FAM84B^low^**	***P*-value**
Characteristics	No.	76	44	
Age (years)				
<60	56	35	21	0.8519
≥60	64	41	23	
Gender				
Male	58	35	23	0.5718
Female	62	41	21	
Tumor size (cm)				
≤3.0	56	30	26	0.0186*
>3.0	64	46	18	
Tumor differentiation				
Well	24	12	12	0.0202*
Moderate	35	18	17	
Poor	61	46	15	
Lymph node status (stage)				
N0	65	48	17	0.0239*
N1	49	24	25	
N2	6	4	2	

Lymph node status (stage) was defined by the AJCC 8^th^ edition (N0: node negative, N1: 1–3 nodes positive for metastatic disease, N2: more than four nodes positive for metastatic disease); *P*-values were derived with Chi-square test or Fisher exact test; *P<0.05.

### FAM84B knockdown affects the proliferation, apoptosis, mitochondrial function, and glycolysis of PDAC cells

**Figure 3:**
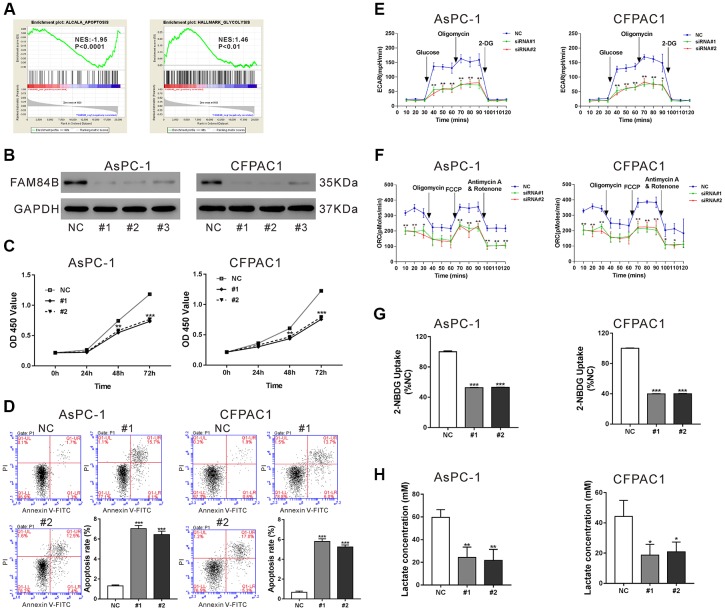
**FAM84B regulates the proliferation, apoptosis, mitochondrial function and glycolysis of PDAC cells.** (**A**) GSEA analysis revealed that FAM84B expression was negatively correlated with apoptosis, but positively correlated with glycolysis in TCGA PDAC dataset. NES: normalized enrichment score. (**B**) Western blotting analysis of FAM84B knockdown efficiency in AsPC-1 and CFPAC1 cell lines. (**C**) CCK-8 proliferation assay indicated that FAM84B knockdown decreased the growth of AsPC-1 and CFPAC1 cells. (**D**) Flow cytometry analysis indicated that FAM84B knockdown induced apoptosis of AsPC-1 and CFPAC1 cells. (**E**) Knockdown of FAM84B significant decreased extracellular acidification rates (ECAR). (**F**) Knockdown of FAM84B significantly decreased oxygen consumption (OCR) in AsPC-1 and CFPAC1 cells. (**G**) Knockdown of FAM84B significantly decreased 2-NBDG uptake. (**H**) Knockdown of FAM84B significantly decreased lactate production. NC: control siRNA; #1, #2, #3: FAM84B siRNA#1, #2, #3. *P<0.05, **P < 0.01 and ***P<0.001 vs. NC.

GSEA analysis revealed a negative correlation between FAM84B expression and apoptosis, and a positive association between FAM84B expression and glycolysis in TCGA dataset (Figure 3A). Thus, we tried to explore the functions of FAM84B in PDAC cells. Lentivirus particles expressing FAM84B shRNAs (#1, #2 and #3) or control shRNA (NC) was transduced into AsPC-1 and CFPAC1 cells, which exhibited relatively higher FAM84B levels as compared to human normal pancreatic epithelial cell line HPDE (Supplementary Figure 1). The western blotting analysis confirmed the knockdown efficiency of all FAM84B shRNAs in both cell lines, and #1 and #2 were selected for the subsequent *in vitro* experiments due to the better knockdown efficiency (Figure 3B).

Next, cell proliferation, apoptosis, and glycolysis were evaluated in AsPC-1 and CFPAC1 cells with FAM84B knockdown. The results from Cell Counting Kit-8 (CCK-8) assay showed that the growth of AsPC-1 and CFPAC1 cells was significantly inhibited at 48 h and 72 h post FAM84B shRNA virus transduction (P <0.01 versus NC, Figure 3C). The results of Annexin V/PI staining plus flow cytometry analysis revealed that the apoptotic ratio of AsPC-1 and CFPAC1 cells was significantly increased at 48 h after transduction with FAM84B shRNA lentiviruses, indicating that FAM84B knockdown induced PDAC cell apoptosis (P <0.001 versus NC, Figure 3D).

The changes of ECAR and ORC following FAM84B knockdown were measured by Seahorse extracellular flux analyzer. As shown in Figure 3E, ECAR values were increased after glucose was added, but the ECAR values of AsPC-1 (#1: 56.3±9.5 mpH/min, #2: 43.7±7.6 mpH/min) and CFPAC1 cells (#1: 53.3±11.7 mpH/min, #2: 43.3±5.1 mpH/min) with FAM84B knockdown were significantly lower than those transduced with NC (AsPC-1: 136.7±12.5 mpH/min, CFPAC1: 127.3±10.7 mpH/min, P <0.01). ECAR values were boosted after inhibition of ATP synthase with oligomycin, and FAM84B knockdown cells showed a significantly weaker increase in ECAR compared to NC cells (P <0.01). After inhibition of ATP synthase with oligomycin, OCR values were remarkably reduced, and OCR values were notably increased with the addition of the mitochondrial membrane uncoupler FCCP in both cell lines (Figure 3F). Interestingly, more prominent changes were observed in NC cells (P <0.05). Moreover, FAM84B knockdown could significantly decrease glucose uptake (P <0.001, Figure 3G) and lactate production (P <0.05, Figure 3H) in both PDAC cell lines. These data indicated that FAM84B knockdown inhibited the proliferation, mitochondrial function, and glycolysis of PDAC cells.

### Effect of FAM84B on tumor progression via regulation of the Wnt/β-catenin pathway

**Figure 4:**
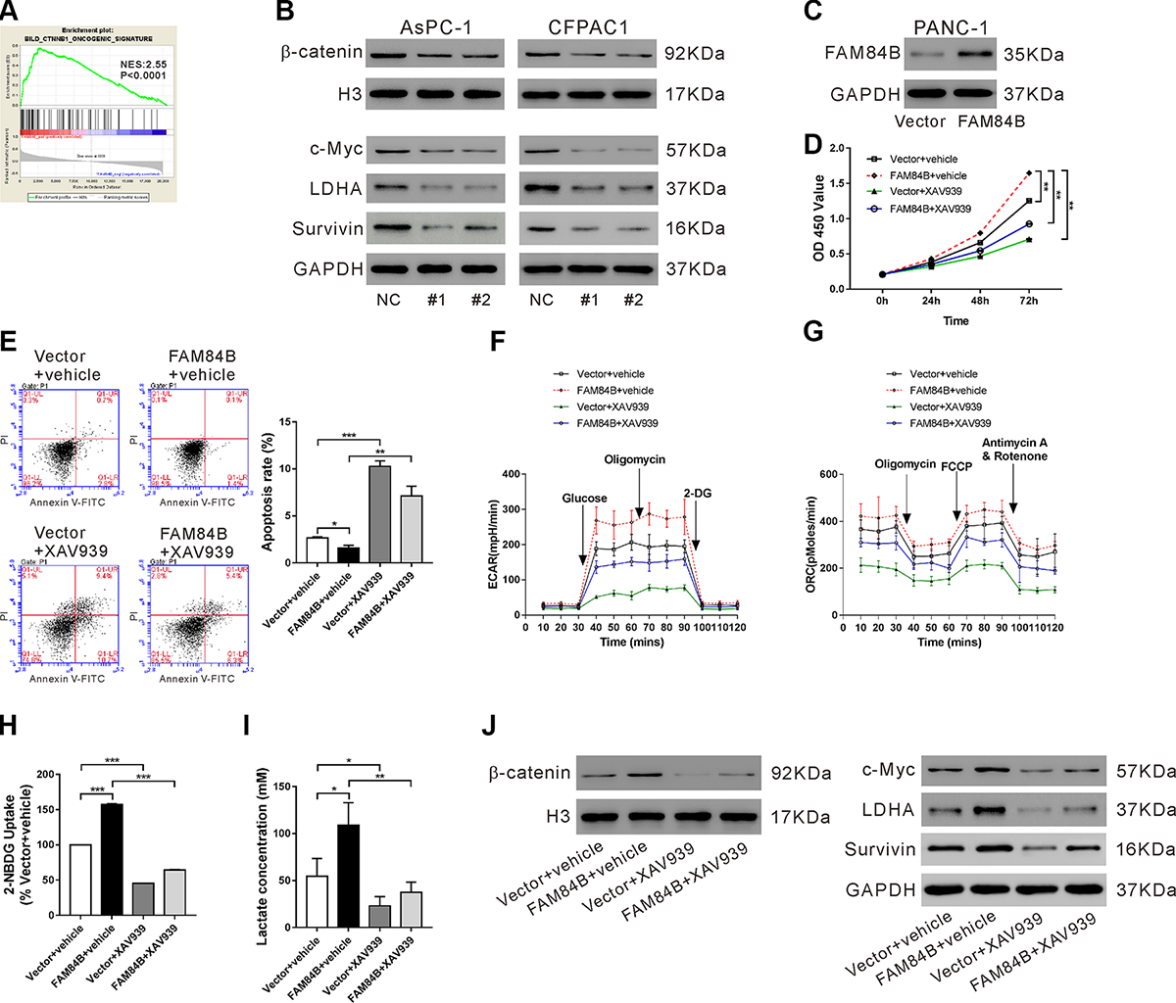
**Effect of FAM84B on tumor progression via regulation of the β-catenin pathway.** (**A**) GSEA analysis revealed that FAM84B expression was positively correlated with CTNNB1 oncogenic signature in TCGA PDAC dataset. NES: normalized enrichment score. (**B**) Western blotting analysis of nuclear β-catenin, and protein expression of c-Myc, LDHA and Survivin in AsPC-1 and CFPAC1 cells with FAM84B knockdown. (**C**) PANC-1 cells were transduced with lentivirus expressing Vector/FAM84B for 48 h and western blotting analysis was performed to assess FAM84B expression. (**D**–**J**) PANC-1 cells were transduced with lentivirus expressing Vector/FAM84B, and then treated with vehicle (DMSO, vehicle) or 10 μM XAV939. Cell growth (**D**), apoptosis (**E**), extracellular acidification rates (ECAR, **F**), oxygen consumption (OCR, **G**), 2-NBDG uptake (**H**) and lactate production (**I**), as well as nuclear β-catenin and the protein levels of c-Myc, LDHA and Survivin (**J**) were detected. **P < 0.01 and ***P<0.001.

Then, we explored the signaling pathways related to FAM84B in PDAC by GSEA analysis and found the CTNNB1 (β-catenin) oncogenic signature was positively correlated with FAM84B in TCGA PDAC dataset (Figure 4A). Nuclear accumulation of β-catenin, an indicator of active Wnt/β-catenin pathway [22], was decreased in AsPC-1 and CFPAC1 cells with FAM84B knockdown (Figure 4B and Supplementary Figure 2A). Additionally, the changes of downstream effectors of the Wnt/β-catenin pathway, c-Myc [22] and Survivin [23] were in line with the alteration of β-catenin nuclear accumulation (Figure 4B). LDHA, a key enzyme of glycolysis and a target of c-Myc [24], was also reduced with FAM84B knockdown.

To demonstrate whether the effect of FAM84B on tumor progression is mediated through the Wnt/β-catenin pathway, PANC-1 cells, which had lower FAM84B expression, were overexpressed with FAM84B and treated with XAV939, an inhibitor for the Wnt/β-catenin pathway [25]. Lentivirus expressing FAM84B obviously enhanced FAM84B expression in PANC-1 cells (Figure 4C). FAM84B overexpressed PANC-1 cells displayed increased proliferation (Figure 4D), ECAR values (Figure 4F), ORC (Figure 4G), glucose uptake (Figure 4H), lactate production (Figure 4I), β-catenin nuclear accumulation (Figure 4J and Supplementary Figure 2B), and expression of c-Myc/LDHA and Survivin (Figure 4J), as well as reduced apoptosis (Figure 4E). XAV939 treatment suppressed the effects of FAM84B overexpression on PANC-1 cells. These data indicated that the Wnt/β-catenin pathway may be involved in the functions of FAM84B on pancreatic carcinogenesis.

### Effect of FAM84B knockdown on tumor progression *in vivo*

**Figure 5:**
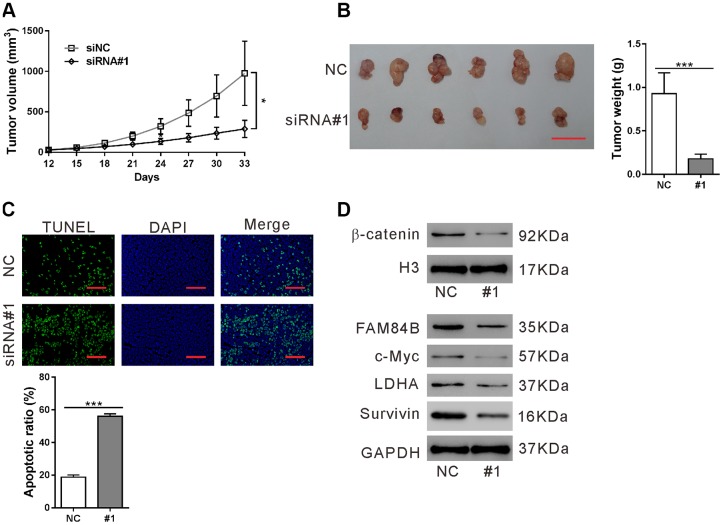
**Effect of FAM84B knockdown on tumor progression *in vivo*.** Nude mice were transplanted with AsPC-1 cells stably expressing control siRNA (NC) or FAM84B siRNA (siRNA#1). (**A**) Tumor volume were recorded for 33 days. (**B**) At 33 days post transplantation, xenografts were collected, photographed and weighed. Scale bar: 2 cm. (**C**) TUNEL assay was performed to assess apoptotic rate in the collected xenografts (magnification scale bar, 100 μm). (**D**) Nuclear β-catenin and the protein levels of c-Myc, LDHA and Survivin were detected. *P < 0.05 and ***P<0.001.

To study the effects of FAM84B knockdown *in vivo*, a xenograft mouse model was constructed by transplantation with AsPC-1 cells expressing control siRNA (NC) or FAM84B siRNA (siRNA#1). The growth of xenografts was significantly suppressed by siRNA#1 (P<0.05, Figure 5A). At 33 days post transplantation, FAM84B knockdown resulted in a decreased tumor weight (P<0.001, Figure 5B), and increased apoptotic rate in xenografts (P<0.001, Figure 5C). Moreover, β-catenin nuclear accumulation and expression of c-Myc/LDHA and Survivin were reduced in xenografts from FAM84B knockdown cells (Figure 5D). These data demonstrated the inhibitory effects of FAM84B siRNA on tumor progression *in vivo*.

### FAM84B expression affects PDAC cell sensitivity to gemcitabine

**Figure 6:**
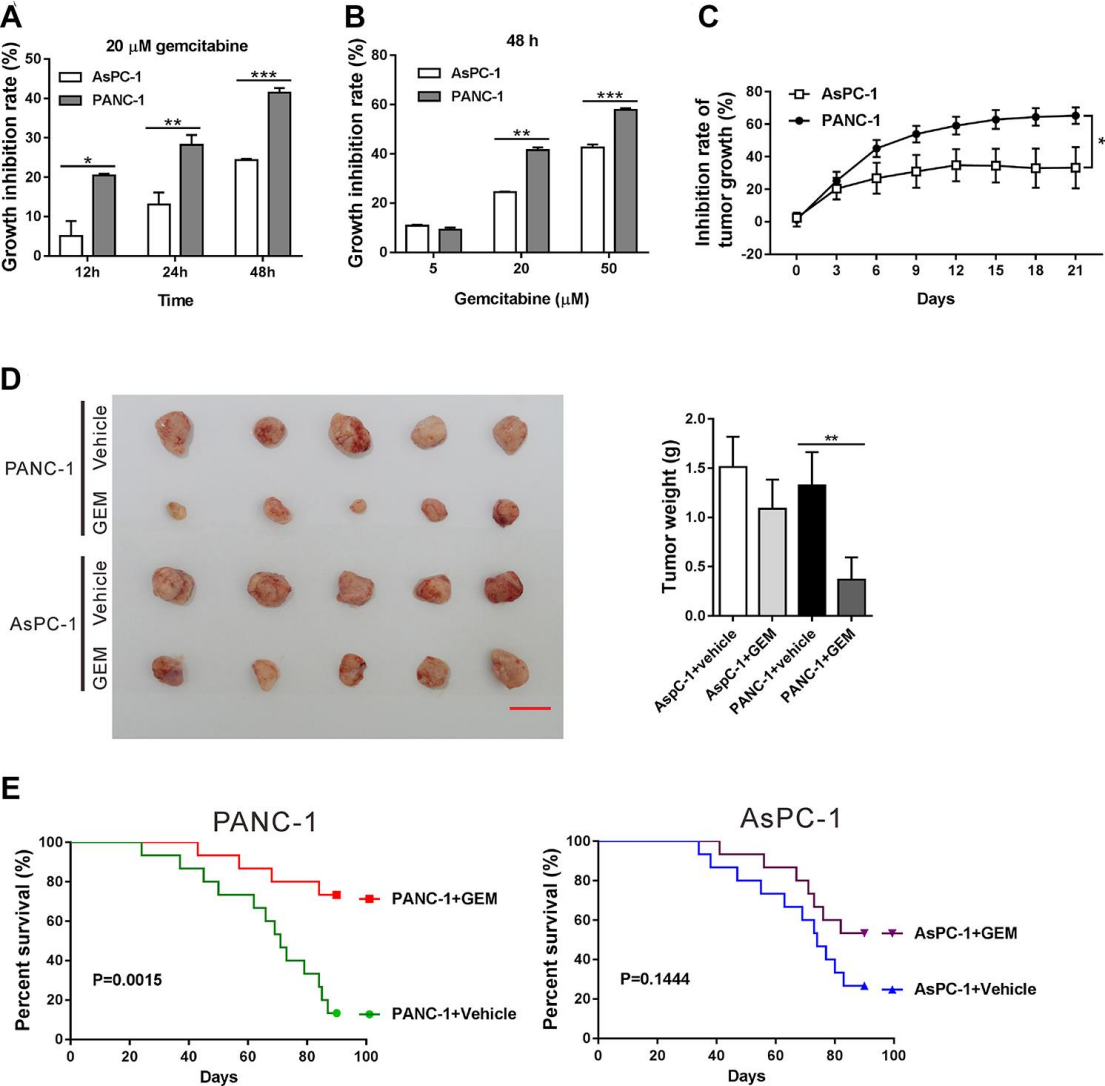
**FAM84B expression affects PDAC cell sensitivity to gemcitabine.** (**A**, **B**) AsPC-1 and PANC-1 cells were exposed to 20 μM gemcitabine or Vehicle (DMSO) for 12, 24 and 48 h (**A**). AsPC-1 and PANC-1 cells were exposed to 5, 20 and 50 μM gemcitabine or Vehicle (DMSO) for 48 h (**B**). CCK-8 assay was performed to assess the inhibition rate of cell proliferation. (**C**–**E**) Nude mice bearing xenografts formed from AsPC-1 and PANC-1 cells were treated with gemcitabine (GEM) or Vehicle (DMSO) (n=20 each group). Inhibition of tumor growth (**C**) were shown. At 21 days after treatment with GEM or Vehicle, 5 mice of each group were sacrificed, and the xenografts were collected, photographed and weighed (**D**). Scale bar: 2 cm. Survival analysis was performed on the remaining mice for 90 days (n=15 each group). *P<0.05, **P<0.01, and ***P<0.001.

We wondered whether FAM84B expression influences PDAC cell sensitivity to gemcitabine. AsPC-1 and PANC-1 cells were exposed to 20 μM gemcitabine or vehicle (DMSO) for 12, 24 and 48 h (Figure 6A), or exposed to 5, 20 and 50 μM gemcitabine or vehicle (DMSO) for 48 h (Figure 6B), and then cell proliferation inhibition rate was assessed with CCK-8 assay. The results showed that PANC-1 cells, which had lower expression of FAM84B (Supplementary Figure 1), were more sensitive to gemcitabine-induced cell proliferation inhibition.

Further, a mouse xenograft model was established with AsPC-1 and PANC-1 cells and then treated with gemcitabine or vehicle (DMSO). As shown in Figure 6C, the growth inhibition rate of xenografts formed from PANC-1 cells was significantly higher than from AsPC-1 cells (P <0.05). At 21 days after treatment, a more prominent reduction in the size and weight of xenografts was observed in PANC-1 cells than AsPC-1 cells (Figure 6D, P <0.01). Moreover, survival analysis suggested that gemcitabine significantly increased the survival rates of mice bearing xenografts formed from PANC-1 cells (Figure 6E, P <0.01), but had little effects on the survival rates of mice bearing xenografts formed from AsPC-1 cells (Figure 6E, P >0.05). Therefore, these results suggested that the anti-tumor effect of gemcitabine in PDAC is more efficient when FAM84B expression was lower.

## DISCUSSION

Accumulated evidence has supported the aberrant expression of FAM84B in carcinogenesis of several human malignancies [13, 15–20]. The current study is the first attempt to investigate the association of FAM84B with PDAC tumorigenesis. By analyzing the publicly available TCGA dataset and our own cohort, we found that FAM84B amplification occurred in PDAC (Figure 1) and that FAM84B expression was elevated in PDAC compared to normal tissues (Figure 2). FAM84B amplification was closely related to its expression. Moreover, the Kaplan-Meier survival curves and log-rank analysis demonstrated that FAM84B amplification and overexpression predicted poor prognosis in PDAC, suggesting the potential clinical value of FAM84B in PDAC.

In the current study, FAM84B knockdown in PDAC cells repressed *in vitro* cell proliferation and *in vivo* tumor growth, which was in line with the findings in ESCC cells [16, 17]. The high rate of aerobic glycolysis or Warburg effect increases lactate production even in the presence of adequate oxygen and is considered to be a key metabolic dependency in tumorigenesis [26, 27]. Our data firstly showed that FAM84B was involved in the aerobic glycolysis of PDAC cells (Figure 3). Together, FAM84B may play an important role during PDAC tumorigenesis by regulating aerobic glycolysis and cell growth.

The Wnt/β-catenin signaling is precisely regulated and plays a critical role in regulating exocrine cell proliferation during pancreatic development [28]. Frequent activation of Wnt/β-catenin signaling has been reported in PDACs [29–31]. Suppression of Wnt/β-catenin signaling decreased cell proliferation and increased apoptosis in PDAC cells [31]. Here, GSEA in TCGA PDAC dataset showed that FAM84B expression was strongly correlated with the CTNNB1 (β-catenin) oncogenic signature (Figure 4A). c-Myc is an important transcription factor and downstream effector of the Wnt/β-catenin pathway. The amplification of c-Myc is also common in PDAC [32]. LDHA, a key enzyme of aerobic glycolysis and a target of c-Myc [24], is elevated in pancreatic cancer and promotes the tumorigenicity of pancreatic cancer cells [33]. Survivin, regulated by the Wnt/β-catenin pathway [23], is an anti-apoptotic factor in PDAC [34]. Here, knockdown of FAM84B decreased the nuclear accumulation of β-catenin, and the expression of c-Myc/LDHA and Survivin *in vitro* (Figure 4B) and *in vivo* (Figure 5D), suggesting that FAM84B may function through regulating Wnt/β-catenin signaling in PDAC. Furthermore, we adopted XAV939, an inhibitor for the Wnt/β-catenin pathway [25] to validate this correlation in FAM84B overexpressed cells. The Wnt/β-catenin pathway inhibition eliminated the effect of FAM84B on proliferation, apoptosis, and glycolysis as well as the expression of c-Myc, which suggests that the Wnt/β-catenin pathway mediates the oncogenic role of FAM84B in PDAC.

Gemcitabine resistance limits the treatment potency and is considered as the main cause of poor prognosis of PDAC [12]. Here, PDAC cells with lower expression of FAM84B were more sensitive to gemcitabine-induced cell proliferation inhibition *in vitro* and *in vivo*. The current study firstly revealed the association between FAM84B expression and gemcitabine resistance and suggested that targeting FAM84B may enhance chemotherapy sensitivity for PDAC (Figure 6).

In conclusion, we observed the amplification and overexpression of FAM84B in PDAC and revealed the its clinical importance. We also found that FAM84B exerted oncogenic roles in PDAC by regulating the Wnt/β-catenin signaling pathway. Moreover, PDAC cells with lower expression of FAM84B were more sensitive to gemcitabine-induced cell proliferation inhibition. Our results may provide new directions for the diagnosis and treatment of PDAC, although some limitations exist. The correlation between the amplification of FAM84B and the chemotherapeutic response of PDAC patients has not been analyzed due to short of clinical samples. The detailed mechanisms of how FAM84B regulated the Wnt/β-catenin signaling pathway is to be explored.

## MATERIALS AND METHODS

### Sample collection

The patients, who were diagnosed with PDAC and underwent surgery at the Department of Gastrointestinal surgery, Changhai Hospital (Shanghai, China) from June 2010 to June 2012, were enrolled in this study after written informed consent was obtained. Cohort 1 and cohort 2 contained 60 and 120 patients, respectively. None of the patients received radiation therapy, chemotherapy, or immunotherapy before surgery. Cancerous tumor specimens from cohort 1 were stored at -80°C and used for copy number variation (CNV) analysis. Specimens from cohort 2 were formalin-fixed and paraffin-embedded and subjected to immunohistochemistry (IHC) staining. The clinicopathological features of cohort 2 were obtained from the medical records and listed in Table 2. This study was approved by the Ethical Community of Changhai Hospital (Shanghai, China).

**Table 2:** Clinicopathological features in patients with PDAC (n=120).

**Characteristics**	**No.**
Age (years)	
<60	56
≥60	64
Gender	
Male	58
Female	62
Tumor size (cm)	
≤3.0	56
>3.0	64
Tumor differentiation	
Well	24
Moderate	35
Poor	61
Lymph node status (stage)	
N0	65
N1	49
N2	6

Lymph node status (stage) was defined by the AJCC 8^th^ edition (N0: node negative, N1: 1–3 nodes positive for metastatic disease, N2: more than four nodes positive for metastatic disease).

### Bioinformatics analysis

The Cancer Genome Atlas (TCGA) pancreatic ductal adenocarcinoma (PDAC) dataset was downloaded from the TCGA website (https://tcga-data.nci.nih.gov/tcga/). The association between the DNA copy number and the mRNA expression of FAM84B was determined by GISTIC analysis. Kaplan–Meier survival curves and log-rank tests were conducted with the R survival package.

### Copy number variation (CNV)

Genomic DNA was isolated from 60 PDAC specimens of cohort 1 with TGuide S32 Magnetic Tissue DNA Kit (TIANGEN, Shanghai, China) following the manufacturer’s protocol. After estimation of DNA concentration with NanoDrop 1000 (Thermo Scientific, Rockford, IL, USA), copy number of *FAM84B* gene was detected by real-time PCR on QX200 Droplet Digital PCR System (Bio-Rad, Richmond, CA, USA) according to the manufacturer’s instructions with ribonuclease P/MRP subunit p40 (RnaseP) as internal control. The following primers and probes were used: FAM84B: primers, 5' -CAGCTCCGGTGCTTGTTG -3' and 5' –AGGTGTAGGCTCGGCAGTCC- 3', target probe: 5'-FAM-TCTACAATCCCGTCCCTCCAGC-BHQ1-3'; RnaseP: primers, 5' – TATGTCTCGCTGTTTCTG -3' and 5' –TGCTTATTACTGCCCTCA - 3', target probe: 5'- CY3-ATTCGTTGCTTTGGTATTCTGTGGA-BHQ -3'.

### IHC staining

The formalin-fixed and paraffin-embedded samples were cut into 4 μm sections. Following de-paraffinization in xylene and rehydration in a series of alcohol, endogenous peroxidase was quenched by incubation with 0.3% hydrogen peroxide for 30 min and antigen retrieval was performed with citrate buffer (pH 6.0) in a high-pressure cooker for 10 min. The sections were probed with rabbit antibody against FAM84B (Proteintech, Chicago, IL, USA) in a moist chamber at 4°C overnight, and then with HRP-labelled secondary antibody. Subsequently, the sections were stained with DAB (3,3-diaminobenzidine) and then counterstained with hematoxylin. The IHC results were evaluated by two experienced pathologists. The staining intensity was scored as 0, negative; 1, weakly positive; 2, moderate positive; 3, strong positive. The percentage of positive cells was graded as 0, negative–10%; 1, 11%-25%; 2, 25-50%; 3, >50%. The Staining Index was calculated with the following formula: Staining Index = staining intensity × percentage of positive staining cells. The case was defined as high expression when the Staining Index is higher than 3.

### Cell lines

The human pancreatic cancer cell lines (AsPC-1, BXPC3, CFPAC1, PANC-1 and SW1990), human normal pancreatic epithelial cell line HPDE, and 293 cells were obtained from Cell Bank of Shanghai Institute of Biochemistry and Cell Biology (Shanghai, China). All the cell lines were cultured in Dulbecco’s Modified Eagle’s Medium (DMEM; Hyclone, Logan, UT, USA) supplemented with 10% fetal bovine serum (FBS; Gibco, Carlsbad, CA, USA) at 37°C with 5% CO2.

### Plasmids and RNA interference

The complementary DNA for human FAM84B was inserted into the pLVX-puro expression vector purchased from Clontech (Palo Alto, CA, USA). shRNA sequences targeting FAM84B (#1: 5’- GCAACCAGGTGGAGAAATT-3’; #2: 5’- GCATCTACCAGAAGAGCTT -3’; #3: 5’- CCGTATATGTGGGTAACTT -3’) and a negative control sequence (NC) were synthesized and inserted into the pLKO.1 vector (Addgene, Cambridge, MA, USA). Sequence analysis was performed to confirm the successful construction of plasmids.

### Virus production

The FAM84B overexpression plasmid vector, FAM84B shRNAs (#1, #2 and #3) or control shRNA (NC) together with the packaging plasmids were transfected into 293 cells using opti-MEM (Invitrogen, Carlsbad, CA, USA) and Lipofectamine 2000 (Invitrogen) following the manufacturer’s instructions. Supernatants containing lentiviruses were collected 48-72h later and used to infect PDAC cell lines.

### Western blotting

To extract total protein, tissue samples and cells were lysed using ice-cold radioimmunoprecipitation (RIPA) lysis buffer supplemented with protease inhibitors (Solarbio, Beijing, China). The protein concentrations were determined using a protein quantitation kit (Thermo Fisher Scientific, Rockford, IL, USA). NE-PER Nuclear and Cytoplasmic Extraction Reagents (Thermo Scientific) were used for nuclear protein extraction according to the manufacturer’s protocol. Total protein or nuclear protein was then subjected to 10% or 15% SDS-PAGE and transferred onto nitrocellulose membranes (Millipore, Bredford, MA, USA). After the non-specific binding was blocked with 5% nonfat dried milk in TBST for 1 h, the membranes were incubated with primary antibodies at 4°C overnight. The following primary antibodies were used: anti-FAM84B, anti-c-Myc, and anti-LDHA were from Abcam (Cambridge, MA, USA), and anti-Survivin, anti-GAPDH, anti-β-catenin, and anti-H3 were obtained from Cell Signaling Technology (Danvers, MA, USA). After washing with TBST and incubation with corresponding HRP-conjugated secondary antibodies (Beyotime, Shanghai, China), the immunoreaction was detected with enhanced chemiluminescence (Millipore).

### Cell counting kit-8 assay

Cell Counting Kit-8 (CCK-8) assay was performed to determine cell proliferation. Cells were plated in 96-well culture plates at a density of 3×10^3^ cells per well, cultured overnight and then treated as indicated. After treating for 0, 24, 48, or 72 h at 37°C with 5% CO2, the cultured medium was replaced with 10% CCK-8 reagent (SAB biotech. College Park, MD, USA) in DMEM for 1 h. Optical density (OD) at 450 nm was determined by a microplate reader.

To assess the effects of gemcitabine on cell proliferation inhibition, AsPC-1 and PANC-1 cultured in 96-well culture plates were exposed to 20 μM gemcitabine (Aladdin, Shanghai, China) or Vehicle (DMSO) for 12, 24 and 48 h, or exposed to 5, 20 and 50 μM gemcitabine or Vehicle (DMSO) for 48 h. The CCK-8 assay was conducted as described above, and cell proliferation inhibition (%) was calculated as follows: Inhibition rate (%) =1– OD_treated_/OD_vehicle_.

### Cell apoptosis assay

Cells were plated in 6-well culture plates at a density of 3×10^5^ cells per well, cultured overnight and then treated as indicated. After treating for 48 h at 37°C with 5% CO2, cells were collected, washed with PBS, and stained with Annexin V-FITC/PI kit (Beyotime) as the manufacturer suggested. The apoptotic rate was analyzed by a flow cytometer (BD Biosciences, Franklin Lakes, NJ, USA).

### Measurement of cellular respiration and glycolytic activity

Extracellular acidification rate (ECAR) and oxygen consumption rate (OCR) were monitored using Seahorse XF96 extracellular flux analyzer (Seahorse Bioscience, North Billerica, MA, USA) according to the manufacturer’s instructions. The cells were plated in microplates at a density of 4×10^4^ cells/well one day before measurement. For examining ECAR, the glyco-stress test kit (Seahorse Bioscience), 10 mM glucose, 2 μM oligomycin, and 50 mM 2-NBDG (2-[N-(7-nitrobenz-2-oxa-1,3-diazol-4-yl) amino]-2-deoxyglucose) were used. For examining OCR, a mito-stress kit, 2 μM Oligomycin, 1.5 μM fluoro-carbonyl cyanide phenylhydrazone (FCCP), and premixed 1 μM antimycin A solution +100 nM rotenone was used.

### Measurement of 2-NBDG uptake and lactate production

The culture medium was collected and subjected to measurement of lactate production by using Lactic Acid Detection Kit (Nanjing Jiancheng Bioengineering Institute, Nanjing, China), while the cells were subjected to 2-NBDG uptake assay. The cells were cultured with glucose-free medium (GIBCO) at 37 °C for 15 min, and then 2-NBDG at a final concentration of 100 μM was added. After culture at 37°C for 45 min, the cells were washed with glucose-free Krebs-Ringer buffer (KRB) and analyzed using flow cytometry. The values of treated groups were normalized to that of control and expressed as percentage of Control.

### Animal experiments

All animal experiments were performed in accordance with procedures approved by the Animal Care Committee of Changhai Hospital (Shanghai, China). Four-week-old BALB/C nude mice weighing 18-20 g were purchased from Shanghai Experimental Animal Center (Shanghai, China) and maintained under specific pathogen-free (SPF) conditions.

To investigate the effects of FAM84B expression level on PDAC cell sensitivity to gemcitabine, a mouse xenograft model was created by transplantation of AsPC-1 and PANC-1 cells into the flank of nude mice (5 × 10^5^ cells per mouse, n=40 per cell line). At 12 days post transplantation, and the mice randomly divided into two groups (n=20 per group) and injected with gemcitabine (50 mg/ kg /day) or Vehicle (DMSO) every three days. Tumor volumes were recorded every three days for 21 days since treatment. At 21 days post treatment, five mice of each group were sacrificed the xenografts were collected and weighed. Survival analysis last 90 days on the remaining mice (n=15 per group).

AsPC-1 cells expressing FAM84B shRNA (siRNA#1) or control shRNA (NC) were subcutaneously transplanted into the flank of nude mice (5 × 10^5^ cells per mouse, n=5). Tumor volumes were recorded every three days. At 33 days post transplantation, the mice were sacrificed the xenografts were collected and weighed. Cell apoptosis in xenografts was assessed by TUNEL assay (Roche, Indianapolis, IN, USA) and protein expression was analyzed by western blotting.

### Statistical analysis

All *in vitro* data were repeated independently at least three times and presented as the mean ± SD (standard deviation). All data were analyzed using the Graphpad Prism software (version 6.0, San Diego, CA, USA). One-way analysis of variance (ANOVA) and two-tailed Student’s t-test were used for statistical analysis. A chi-square test or Fisher exact test was conducted to analyze the association between FAM84B expression and clinicopathological features. Kaplan-Meier method and the log-rank test was employed for overall survival analysis. P value less than 0.05 was considered to indicate statistical significance.

## Supplementary Material

Supplementary Figures
