# Supplementary material for: FAM84B, amplified in pancreatic ductal adenocarcinoma, promotes tumorigenesis through the Wnt/β-catenin pathway
Source: Aging (Albany NY). 2020 Apr 14;12(8):6808–22. doi: 10.18632/aging.103044 (PMC7202512; doi:10.18632/aging.103044)
Supplement: Supplementary Figures [file aging-12-103044-s001..pdf]

SUPPLEMENTARY FIGURES

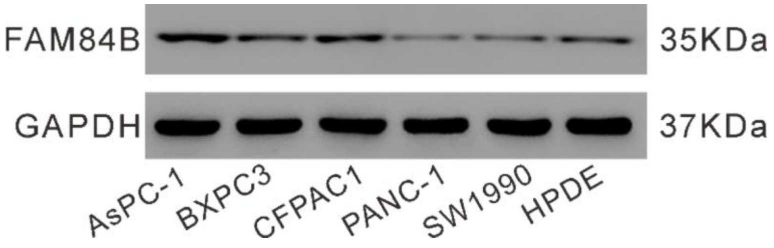

Supplementary Figure 1. Western blotting analysis of FAM84B protein expression in 5 pancreatic cancer cell lines and HPDE cells.

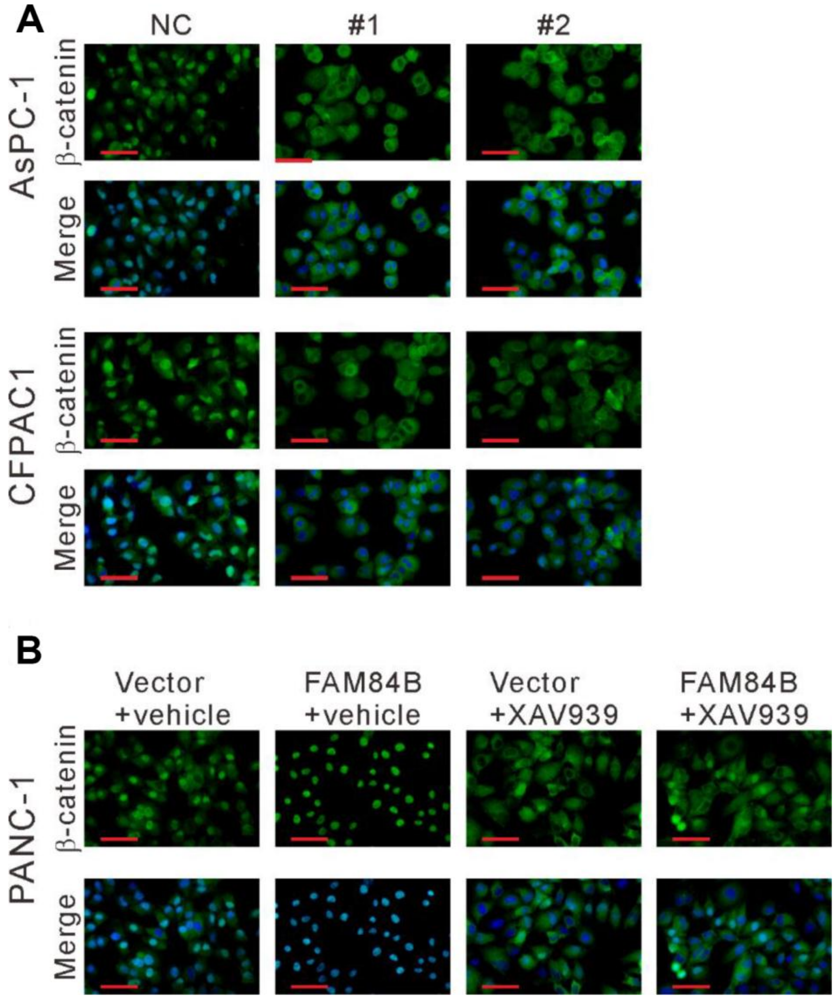

Supplementary Figure 2. Immunofluorescence analysis of β-catenin (green) localization. (**A**) AsPC-1 and CFPAC1 cells with FAM84B knockdown. (**B**) PANC-1 cells were transduced with lentivirus expressing Vector/FAM84B, and then treated with vehicle (DMSO, vehicle) or 10 μM XAV939. After fixation and permeabilization, the cells were incubated with rabbit anti-β-catenin (Abcam) and then with Alexa Fluor 488 goat anti-rabbit secondary antibody (Beyotime). DAPI (blue) was used to stain nuclei (magnification scale bar, 50 μm).
